# Influence of Diabetes Knowledge, Self-Stigma, and Self-Care Behavior on Quality of Life in Patients with Diabetes

**DOI:** 10.3390/healthcare10101983

**Published:** 2022-10-10

**Authors:** Sung Eun Cho, Myoungjin Kwon, Sun Ae Kim

**Affiliations:** 1 Division of Nursing, Chungnam National University Hospital, Daejeon 35015, Korea; 2 Department of Nursing, Daejeon University, Daejeon 34520, Korea; 3 Department of Nursing, Korea National University of Transportation, Cheongju 27909, Korea

**Keywords:** diabetes, diabetes knowledge, self-stigma, self-care behavior, quality of life

## Abstract

Globally, almost 9.3% of the population aged 20–80 years have been diagnosed with diabetes making diabetes management a global health problem beyond specific regions or races. This study aimed to determine the effect of diabetes knowledge, self-stigma, and self-care behavior on the quality of life of patients with diabetes. This descriptive research study evaluated 180 patients receiving diabetes treatment at the outpatient Department of Endocrinology at C University Hospital. Data were collected between 30 July 2019, and 30 August 2019. The study variables were general patient characteristics, disease-related characteristics, quality of life, diabetes knowledge, self-stigma, and self-care behavior. Factors affecting the quality of life were analyzed by hierarchical regression. Self-stigma (β = −0.298), monthly income (β = 0.270), and self-care behavior (β = 0.140) significantly affected the quality of life, in that order. The higher the self-stigma, the lower the quality of life, and the higher the monthly income and the level of self-care behavior, the higher the quality of life. A psychosocial support program to positively change the attitude toward diabetes is needed to improve the quality of life among patients with diabetes.

## 1. Introduction

Globally, approximately 9.3% of the population aged from 20 years to 80 years have been diagnosed with diabetes [1]. According to The International Diabetes Federation (IDF) Diabetes Atlas, 10th edition, worldwide, one in 10 people have diabetes, and if this trend continues, 783 million people will be diabetic by 2045 [2]. Therefore, diabetes management is a global health problem beyond specific regions or races. In South Korea, diabetes is the sixth leading cause of death [3]. Knowledge related to diabetes and its management is crucial for successful diabetes management [4]. Diabetes is a chronic disease that requires continuous medical management, and continuous self-management education can reduce the risk of acute and chronic complications [5] and the quality of life.

Self-stigma, which has been rarely addressed in patients with diabetes, might be a major influencing factor on the emotional status of patients with diabetes. Stigma refers to the labeling of individuals to distinguish them from others or to devalue them so that discrimination occurs [6]. Diabetes diagnosis is associated with negative stereotypes because many people perceive diabetes as a lifestyle-related disease [7]. In particular, the self-stigma of diabetic patients can affect self-management and blood sugar control, and the occurrence of stigma is high [8]. Moreover, when blood sugar is not controlled, self-stigma is reinforced and self-management motivation is decreased [9]. Most patients with diabetes experience stigma and feel criticized by others [10], and this is negatively associated with their quality of life [11]. Therefore, the relationship between self-stigma and quality of life needs to be investigated in patients with diabetes.

Diabetes can be accompanied by several complications but certain complications can be prevented with proper self-care [12]. Self-care behaviors in patients with diabetes include diet, physical activity, blood sugar control, drug use, problem solving, coping, and risk reduction. Diabetes management is very important, and more than 98% of diabetes management is self-management [13]. Diabetes management is closely related to self-care. The American Association of Diabetes emphasizes that diabetes’ self-care behavior improves the quality of life of patients with diabetes and is related to the management of complications [14]. However, another study reported that there was no correlation between diabetes self-management and quality of life, showing inconsistent results [15].

Therefore, this study aimed to examine the relationship among diabetes knowledge, self-stigma, and self-care behavior as important variables influencing the quality of life of patients with diabetes. In addition, this study aimed to present basic data for nursing intervention to improve the quality of life of patients with diabetes.

## 2. Materials and Methods

### 2.1. Study Design and Participants

This descriptive research study evaluated the patients receiving diabetes treatment at the outpatient department of endocrinology at Chungnam National University Hospital. The inclusion criteria were age ≥18 years; diagnosed with diabetes for more than 6 months; use of active insulin or oral hypoglycemic agent; and ability to communicate, understand, and respond to the questionnaire. The required number of patients was calculated using the G*power (ver. 3.1.9.2) program [16]. At least 172 patients were needed to achieve a median effect size of 0.15, significance level of 0.05, power of 0.95, and 10 variables. Considering the dropout rate of about 10%, a total of 192 people were surveyed, and the results of 180 people’s responses to the survey (93.8%) were analyzed excluding incomplete responses. Data were collected from 30 July 2019 to 30 August 2019.

This study was approved by the Institutional Review Board of Daejeon University (1040647–20190–HR–004-03) and was conducted according to the tenets of the Helsinki Declaration.

### 2.2. Study Variables

#### 2.2.1. General and Disease-Related Characteristics

The general characteristics were gender, age, education level, marital status, religion, job, drinking, smoking, and monthly income. Disease-related characteristics included the duration of diabetes, diabetes type, diabetes medication, number of insulin injections, diabetes education experience, diabetes hospitalization experience, number of self-monitoring blood glucose measurements, experience with hypoglycemia within the last 3 months, number of glycated hemoglobin within the last 3 months, diabetes complications, and health problems other than diabetes.

#### 2.2.2. Diabetes Knowledge

Diabetes knowledge was measured using a tool developed by the Michigan Diabetes Research and Education Center [17] and general knowledge items of the Diabetes Knowledge Test translated into Korean by Choi [18]. A total of 14 questions were asked, and responses were allotted 1 point if correct and 0 point if incorrect. The total score ranged from 0 to 14 points and a higher score indicated higher knowledge about diabetes.

#### 2.2.3. Self-Stigma

Self-stigma was assessed using the self-stigma scale in people with diabetes by Seo and Song [19]. The scale includes 16 questions and consists of 4 domains: social atrophy factors (4 questions), self-value lowering factors (4 questions), negative emotional factors (4 questions), and relative incompetence factors (4 questions). A high score means that the degree of stigma is high. In this study, Cronbach’s ɑ was 0.92.

#### 2.2.4. Self-Care Behavior

Self-care behavior was measured using the self-management measurement tool developed by Kim [20]. It includes 20 questions and consists of five areas: dietary practice (7 questions); drug use (3 questions); physical exercise (2 questions); blood sugar test (3 questions); and general health-care (5 questions). A higher score indicates a higher degree of self-care behavior. In Kim’s study [19], the Cronbach’s ɑ was 0.85, while it was 0.79 in the current study.

#### 2.2.5. Quality of Life

Quality of life was assessed using the Korean version of the World Health Organization Quality of Life simple scale (WHOQOL-BREF) developed by Min et al. [21]. It consists of 26 questions in 4 domains: physical health (7 questions); psychological domain (6 questions); social domain (3 questions); living environment domain (8 questions); and overall quality of life (2 questions). A higher score indicates higher quality of life. In the study of Min et al. [21], the Cronbach’s ɑ was 0.89, while it was 0.92 in the current study.

### 2.3. Statistical Analysis

The general and disease-related characteristics of the subjects were presented as the frequency and percentage. A *t*-test or ANOVA was used to determine the difference in quality of life according to general characteristics and disease-related characteristics. The correlation among the subjects’ diabetes knowledge, self-stigma, self-care behavior, and quality of life was analyzed with Pearson’s correlation coefficient. The factors affecting the quality of life of the subjects were analyzed by hierarchical regression. All statistical analyses were performed using the IBM SPSS 25.0 program (IBM Corp., Armonk, NY, USA). A *p*-value of <0.05 was considered statistically significant.

## 3. Results

### 3.1. Differences in Quality of Life According to General Characteristics

There were significant differences in quality of life according to sex, education level, occupation, drinking status, and monthly income. Table 1 shows the differences in quality of life by patient characteristics. The quality of life of men was higher than that of women (*t* = 3.60, *p* < 0.001) and the quality of life was higher for those with a college degree or higher than those with less than that (F = 3.16, *p* = 0.026). The quality of life of those with a job was higher than that of those without (*t* = 2.34, *p* = 0.020). The quality of life was 91.25 for the drinkers and 86.29 for the non-drinkers, indicating a significant difference between these two groups (*t* = 2.28, *p* = 0.023). There were also differences in quality of life according to monthly income (F = 1.62, *p* = 0.030).

### 3.2. Differences in Quality of Life According to Disease-Related Characteristics

Table 2 shows the differences in the quality of life according to disease-related characteristics. There was a significant difference in the quality of life according to the glycated hemoglobin within 3 months (F = 1.99, *p* < 0.001), diabetes complications (*t* = 2.36, *p* = 0.019), and health problems other than diabetes (*t* = 2.44, *p* = 0.016), with patients with these conditions showing a lower quality of life.

### 3.3. Correlation between Diabetes Knowledge, Self-Stigma, Self-Care Behavior, and Quality of Life

Table 3 shows the correlations between the subjects’ diabetes knowledge, self-stigma, self-care behavior, and quality of life. Quality of life had a significant positive correlation with diabetes knowledge (r = 0.15, *p* < 0.05) and a significant negative correlation with self-stigma (r = −3.72, *p* < 0.001). Self-care behavior had a significant positive correlation with diabetes knowledge (r = 0.29, *p* < 0.001).

### 3.4. Influencing Factors of Quality of Life

To understand the explanatory power of variables affecting the quality of life of patients with diabetes, a hierarchical multiple regression analysis using three models (in model 1, only general characteristics were input, in model 2, health-related characteristics were additionally added to model 1, and in model 3, diabetes knowledge, self-stigma, and self-care behavior were additionally added to model 2) was performed. Before the hierarchical regression analysis, the tolerance limit and variance inflation factor (VIF) were assessed to check multi-collinearity among the variables, and the dispersion expansion coefficient was ≤10, indicating that there was no multi-collinearity. The Durbin–Watson statistic, which indicated the mutual independence between the residuals, was 1.754, which was close to 2. This indicated that the residuals were mutually independent. The influencing factors of quality of life are shown in Table 4.

In Model 3, the explanatory power of quality of life with the addition of the independent variable increased to 27.4% (F = 7.75, *p* < 0.001). Self-stigma (β = −0.298), monthly income (β = 0.270), and self-care behavior (β = 0.140) were found to have a significant effect on the quality of life, in that order. The higher the self-stigma, the lower the quality of life, and the higher the monthly income and the level of self-care behavior, the higher the quality of life. Diabetes knowledge did not show any significant influencing factor on the quality of life.

## 4. Discussion

The current study found differences in the quality of life according to the general characteristics and disease-related characteristics.

As a result of this study, drinkers had a higher quality of life than non-drinkers. In contrast, a previous study [22], reported a lower quality of life in those who consume alcohol. Moderate alcohol consumption has a net protective effect on lowering mortality in diabetic patients, but in regions with high alcohol consumption, excessive alcohol consumption has a negative effect on mortality and morbidity [23]. This indicated that the more important factor affecting the health of patients with diabetes is the amount of alcohol consumed, and the importance of management in minimizing alcohol intake in subjects with diabetes has been reported [24]. However, the current study did not investigate the amount of alcohol intake, and thus, it was not possible to confirm the difference in the effect on risk according to the amount of alcohol consumed.

The current study found a lower quality of life among patients with diabetes with complications, with a higher number of health problems associated with a lower quality of life. These results are consistent with the results of many previous studies [25].

Correlation analysis of the variables showed that the knowledge of diabetes and self-care behavior were positively correlated with the quality of life, while self-stigma was negatively correlated. Previous studies reported poor health outcomes in patients with diabetes with insufficient self-care behavior and with insufficient knowledge [26]. Quality of life analysis using the WHOQOL-BREF tool in a previous study showed that the higher the knowledge, the higher the quality of life [27]. Our results support these previous findings. Regression analysis in the current study showed that self-stigma, monthly income, and self-care behavior influenced the quality of life of patients with diabetes. Particularly, self-stigma affected the quality of life. The higher the self-stigma, the lower the quality of life. These results are consistent with the results of previous studies indicating that the higher the self-stigma, the lower the quality of life of patients with diabetes [28]. This indicates that the assessment and control of self-stigma are necessary among patients with diabetes. Moreover, most diabetic patients experience stigma and feel criticized by others [10]. To reduce self-stigma, it is necessary to develop a psychosocial support program to positively change the attitude toward diabetes along with an in-depth analysis of the factors affecting self-stigma.

Monthly income was found to have a positive effect on the quality of life. The higher the monthly income, the higher the quality of life. In a previous study on the factors affecting the quality of life in patients with type 1 and 2 diabetes, poor economic status was a predictor of low quality of life [29]. Income is a very important factor, and it has been reported that diabetes morbidity rates in regions with high incomes are stable or declining [2]. A study comparing the prevalence of diabetes by income level also found that with the high-income group as a reference, the middle income, near poor, and poor income groups had higher prevalence rates (40.0%, 74.1%, and 100.4%, respectively) [30]. Low-income patients with diabetes have to choose between spending money on blood sugar monitoring equipment or drugs for disease control and living expenses such as food and electricity bills. Health care may not be prioritized. Multilateral support for health management is needed for patients with diabetes from the socio-economically disadvantaged class. Self-care behavior was also found to have a positive effect on the quality of life. Diabetes self-management consists of healthy eating, regular physical activity, smoking cessation, and maintaining an appropriate weight [2]; ultimately, self-management acts as a factor influencing the quality of life of diabetic patients. Therefore, it is necessary to provide public health policy support from the vulnerable groups for government-level management [2]. The higher the self-care behavior, the higher the quality of life, consistent with a previous study [31].

The limitation of this study is that as the study participants were recruited from those receiving treatment in the Endocrinology Department of one University Hospital, the findings may have limited generalizability. However, this study is meaningful in that it provided basic data on the factors affecting the quality of life of the patients with diabetes. Future studies that can prove a more reliable causal relationship using longitudinal data are needed.

## 5. Conclusions

As a result of this study, self-stigma, monthly income, and self-care behavior were found to have a significant effect on the quality of life. A psychosocial support program to positively change the attitude toward diabetes is needed to improve the quality of life of patients with diabetes. An in-depth analysis of the factors affecting self-stigma is also needed to reduce self-stigma among patients with diabetes. In addition, it is necessary to prepare support measures for patients from the socio-economically disadvantaged class and to provide comprehensive interventions and evaluations to enhance self-care behavior.

## Figures and Tables

**Table 1:** Comparison of quality of life according to general characteristics (*n* = 180).

Variables	Categories	*n* (%)/Mean ± SD	Mean ± SD	*t*/F	*p*
Sex	Male	94 (52.2)	91.00 ± 12.62	3.60	<0.001
	Female	86 (47.8)	84.08 ± 13.09		
Age, years	≤49	37 (20.6)	86.32 ± 12.69	1.62	0.200
	50–69	81 (45.0)	89.65 ± 14.26		
	≥70	62 (34.3)	85.95 ± 12.07		
Education level	≤Elementary school	31 (17.2)	84.23 ± 14.83	3.16	0.026
	Middle school	28 (15.6)	83.68 ± 13.62		
	High school	74 (41.1)	88.08 ± 13.25		
	≥College	47 (26.1)	91.77 ± 10.92		
Marital status	Married	164 (91.1)	87.75 ± 13.08	0.17	0.858
	Single	16 (8.9)	87.13 ± 15.51		
Religion	Yes	81 (45.0)	87.02 ± 12.94	−0.61	0.542
	No	99 (55.0)	88.24 ± 13.57		
Job	Yes	83 (46.1)	90.17 ± 12.75	2.34	0.020
	No	97 (53.9)	85.58 ± 13.41		
Alcohol drinking	Yes	51 (28.3)	91.25 ± 11.35	2.28	0.023
	No	129 (71.7)	86.29 ± 13.74		
Smoking	Yes	30 (16.7)	87.23 ± 13.40	−0.20	0.836
	No	150 (83.3)	87.79 ± 13.29		
Monthly income (KPW10,000)		182.11 ± 194.09	87.69 ± 13.27	1.62	0.030

**Table 2:** Comparison of quality of life according to disease-related characteristics (N = 180).

Variables	Categories	*n* (%)/Mean ± SD	Mean ± SD	*t*/F	*p*
Duration of illness (years)		11.60 ± 9.33	87.69 ± 13.27	0.73	0.820
Type of diabetes	Type 1	11 (6.1)	84.55 ± 16.02	−0.81	0.418
	Type 2	169 (93.9)	87.90 ± 13.10		
Medication types	Oral medication	115 (63.9)	88.26 ± 12.55	0.45	0.639
	Oral medication + insulin injection	34 (18.9)	87.59 ± 11.33		
	Insulin injection	31 (17.2)	85.71 ± 17.48		
Number of insulin injections(/day)	0	115 (63.9)	88.26 ± 12.55	0.30	0.734
	1–2	44 (24.4)	86.45 ± 13.75		
	3–4	21 (11.7)	87.69 ± 13.27		
Diabetes-related education experience	Yes	123 (68.3)	88.37 ± 12.44	1.00	0.314
	No	57 (31.7)	86.23 ± 14.92		
Diabetes-related hospitalization experience	Yes	36 (20)	85.19 ± 14.63	−1.26	0.207
	No	144 (80)	88.32 ± 12.89		
Number of self-monitoring blood sugar measurements (/day)	<1	82 (45.5)	88.20 ± 13.10	0.16	0.846
	1–2	55(30.6)	87.69 ± 11.69		
	≥3	43 (23.9)	86.74 ± 15.57		
Low blood sugar experience	Yes	61 (33.9)	88.48 ± 12.97	−1.10	0.269
	No	119 (66.1)	87.69 ± 13.27		
Glycated hemoglobin (mg/dl)		7.51 ± 1.55	83.08 ± 12.84	2.36	0.019
Diabetes complication	Yes	36 (20)	88.85 ± 13.17	2.36	0.019
	No	144 (80)	86.22 ± 13.22		
Health problems other than diabetes	Yes	130 (72.2)	91.54 ± 12.74	2.44	0.016
	No	82 (27.8)	91.54 ± 12.74		

**Table 3:** Means, standard deviations and correlations between variables. (*n* = 180).

Variables	Mean (SD)	Diabetes Knowledge	Self-Stigma	Self-Care Behavior	Quality of Life
		r(*p*)	r(*p*)	r(*p*)	r(*p*)
Diabetes knowledge	8.42 (2.48)	1			
Self-stigma	37.33 (12.96)	0.03 (0.623)	1		
Self-care behavior	69.61 (12.22)	0.29 (<0.001)	0.05 (0.48)	1	
Quality of life	87.69 (13.27)	0.15 (<0.05)	−3.72 (<0.001)	0.13 (0.078)	1

**Table 4:** Hierarchical multiple linear regression analysis for quality of life (N = 180).

	Variables		Model 1			Model 2			Model 3		
			β	*t*	*p*	β	*t*	*p*	β	*t*	*p*
General characteristics	Sex (male)	Female	−0.152	−2.00	0.046	−0.160	−2.14	0.033	−0.100	−1.35	0.176
	Education level (≤elementary school)	Middle school	−0.028	−0.31	0.757	−0.027	−0.30	0.763	−0.004	−0.04	0.967
		High school	0.053	0.52	0.598	0.005	0.04	0.962	0.027	0.27	0.780
		≥College	0.075	0.73	0.466	0.008	0.07	0.937	0.013	0.13	0.896
	Job (yes)	No	0.044	0.54	0.585	0.038	0.47	0.638	0.042	0.54	0.587
	Alcohol drinking (yes)	No	−0.057	−0.76	0.446	−0.040	−0.53	0.593	−0.069	−0.96	0.334
	Monthly income		0.294	3.48	0.001	0.281	3.38	0.001	0.269	3.43	0.001
Disease-related characteristics	Diabetes complication (yes)	No				0.090	1.22	0.223	0.042	0.57	0.564
	Health problems other than diabetes (yes)	No				0.114	1.59	0.112	0.106	1.55	0.121
	Glycated hemoglobin					−0.147	−2.10	0.037	−0.081	−1.20	0.229
Diabetes knowledge									0.079	1.08	0.281
Self-stigma									−0.301	−4.26	<0.001
Self-care behavior									0.139	1.99	0.048
F(*p*)			4.92 (<0.001)			4.53 (<0.001)			5.87 (<0.001)		
R (Adj. R^2^)			0.167 (0.133)			0.212 (0.165)			0.315 (261)		

## Data Availability

Not applicable.
